# Mutagenesis as a Tool in Plant Genetics, Functional Genomics, and Breeding

**DOI:** 10.1155/2011/314829

**Published:** 2012-01-22

**Authors:** Per Sikora, Aakash Chawade, Mikael Larsson, Johanna Olsson, Olof Olsson

**Affiliations:** ^1^Department of Plant and Environmental Sciences, Göteborg University, 40530 Göteborg, Sweden; ^2^CropTailorAB, Erik Dahlbergsgatan 11A, 41126 Göteborg, Sweden; ^3^Department of Chemical and Biological Engineering, Chalmers University of Technology, 41296 Göteborg, Sweden

## Abstract

Plant mutagenesis is rapidly coming of age in the aftermath of recent developments in high-resolution molecular and biochemical techniques. By combining the high variation of mutagenised populations with novel screening methods, traits that are almost impossible to identify by conventional breeding are now being developed and characterised at the molecular level. This paper provides a comprehensive overview of the various techniques and workflows available to researchers today in the field of molecular breeding, and how these tools complement the ones already used in traditional breeding. Both genetic (Targeting Induced Local Lesions in Genomes; TILLING) and phenotypic screens are evaluated. Finally, different ways of bridging the gap between genotype and phenotype are discussed.

## 1. Introduction

Plant breeding began as early as 10,000 BC during the Neolithic revolution, when tribes of hunter-gatherers started their shift towards a sedentary and agrarian society [1]. Domestication of crop plants seems to have taken place simultaneously in several subtropical regions, across central Africa, western South America, southeast Asia, and the Mediterranean during this period [2]. It is still a subject of discussion whether early attempts at domestication were consciously guided or random, although cave paintings at the Lascaux cave in France and Altamira in Spain as well as in other places show that early man was conscious of the life cycle and nature around him. The first experiments with plant breeding were most likely limited to selecting the most viable specimens from each harvest for subsequent sowing [3], which nevertheless had a profound impact on crop yield. This selection also altered the plants in new ways, since human selection was in practise often opposite to natural selection [4]. It was realised early, that domesticated plants were not to be considered “natural” and Charles Darwin coined the term “artificial selection” in 1859 to emphasise the difference between selection in nature and man-made selection [5]. He then further elaborated on the subject in a separate book published in 1868 [6]. Systematic selection has, over the years, now changed the domesticated plants to the point where the wild relatives of crop plants often are classified in completely different taxa. The greater yields from the domesticated crops, allowed for an increased human population density, formation of communities, and work specialization in areas other than food production within those communities. The move from foraging to agriculture also brought many negative consequences for humankind, including new infectious diseases and epidemics caused by the increased population density and trade, coupled with a decrease in food diversity [2]. Still, it is safe to say that plant breeding is the very basis of our modern civilization.

Since human demand for good traits and yield is very high, only a small fraction of the world's approximately 200,000 plant species have, through history, survived the rigorous scrutiny of the domestication process. Around 3,000 species may have at some point been used for food, feed, spices, and materials but only as few as around 200 have ultimately been completely domesticated. Today, humankind is relying solely on 15–20 species for the entire world food production [7, 8].

## 2. Mutagenesis and TILLING

During crop evolution there has been a continuous reduction in genetic diversity as breeders have increasingly focused on so-called “elite” cultivars. This genetic erosion eventually became a bottleneck and various techniques to induce mutations and artificially increase variation emerged in the middle of the last century [9]. Initially, X-ray radiation was used as a mutagen since it was readily available to researchers. In 1927, Muller showed that X-ray treatment could increase the mutation rate in a *Drosophila* population by 15,000% [10], and a year later, Stadler observed a strong phenotypic variation in barley seedlings and sterility in maize tassels after exposure to X-rays and radium [11, 12]. Later, more sophisticated techniques such as gamma and neutron radiation were developed at newly established nuclear research centers. During and directly following the Second World War, radiation-based techniques were complemented by chemical mutagens that were less destructive, freely available, and easier to work with. Pioneer work in this area was performed by Auerbach and others, who demonstrated an increased mutation frequency in *Drosophila* following exposure to mustard gas (War Gas) [13, 14]. A few years later, this work was followed by the discovery of methane-sulphonates and other chemical mutagens, which are still in use today [15].

The goal in mutagenesis breeding is to cause maximal genomic variation with a minimum decrease in viability. Among the radiation-based methods, *γ*-ray and fast neutron bombardment now supersedes X-ray in most applications. Of these, *γ*-ray bombardment is less destructive causing point mutations and small deletions whereas fast neutron bombardment causes translocations, chromosome losses, and large deletions. Compared to chemical mutagens, both types of radiation cause damage on a larger scale and severely reduces viability [16, 17].

Chemical mutagens have gained popularity since they are easy to use, do not require any specialised equipment, and can provide a very high mutation frequency. Compared to radiological methods, chemical mutagens tend to cause single base-pair (bp) changes, or single-nucleotide polymorphisms (SNPs) as they are more commonly referred to, rather than deletions and translocations. Of the chemical mutagens, EMS (ethyl methanesulfonate) is today the most widely used. EMS selectively alkylates guanine bases causing the DNA-polymerase to favor placing a thymine residue over a cytosine residue opposite to the O-6-ethyl guanine during DNA replication, which results in a random point mutation. A majority of the changes (70–99%) in EMS-mutated populations are GC to AT basepair transitions [18, 19]. Mutations in coding regions can be silent, missense or nonsense. In noncoding regions, mutations can change promoter sequences or other regulatory regions, resulting in up- or downregulation of gene transcription. Aberrant splicing of mRNA, altered mRNA stability and changes in protein translation may also occur as a result of mutagenesis.

Other mutagens such as sodium azide (Az) and methylnitrosourea (MNU) are also used and often combined into an Az-MNU solution. Genetically, Az-MNU predominantly causes GC to AT shifts, or AT to GC shifts. Thus, contrary to EMS, a shift can happen in either direction [18]. All three chemical mutagens are, as can be expected, strongly carcinogenic and should be handled with extreme care. Unlike EMS, MNU is both sensitive to shock and unstable above 20°C making it complicated to work with. In contrast to EMS and MNU, which are both liquid, Az is a solid dust in its ground state and the additional step of first dissolving the acutely toxic and volatile substance before application makes it less attractive to handle.

Through the years, mutagenesis has generated a vast amount of genetic variability and has played a significant role in plant breeding programs throughout the world. Records maintained by the joint FAO/IAEA Division in Vienna show that 2965 crop cultivars, with one or more useful traits obtained from induced mutations, were released worldwide during the last 40 years [20]. Notable examples are several wheat varieties (e.g., durum wheat used in pasta), barley including malting barley, rice, cotton, sunflower, and grapefruit, resulting in an enormous positive economic impact.

During the last decade, the use of chemically induced mutagenesis has had a renaissance with the development of TILLING (Targeting Induced Local Lesions in Genomes) technology. In TILLING, mutagenesis is complemented by the isolation of chromosomal DNA from every mutated line and screening of the population at the DNA level using advanced molecular techniques.

As in conventional mutagenesis, TILLING seeds are exposed to a strong mutagenic compound, which introduces random mutations across the entire genome. However, extra care is taken to achieve mutation saturation in the target genome. Before creating the TILLING population, most researchers therefore start by establishing a “kill curve” using their mutagen of choice where concentration is plotted against seed survivability. A general rule of thumb is to aim for a 30–80% survival rate [21, 22]. After mutagenesis, the seeds (M_1_) are planted and allowed to self-fertilise and produce a new generation of seeds (M_2_). Typically, one seed from each line is sown to produce the M_2_ population and, DNA is isolated from every single M_2_ plant.

Provided the number of mutations per genome is high enough and the size of the population is large enough, it is likely that a mutated allele of all genes in the genome exists somewhere in the population. To determine the optimal size of a particular TILLING population, the ploidy of the target crop has to be considered. There seems to be a strong correlation between the ploidy level and the induced mutation frequency. It has been shown that a mutation frequency as high as one mutation per 25 Kb can be introduced in hexaploid plants such as oat and wheat without killing the plant or making it infertile, while the maximum mutation frequency of diploid plants such as rice and barley is much lower (Table 1). Therefore, a hexaploid TILLING population seldom needs to exceed 5000 individual lines. Diploid populations, on the other hand, often need to be in the range of tens of thousands [22, 23].

Since TILLING in plants is a large and time-consuming project, it is advisable to consider the logistics of TILLING before performing the mutagenesis. Harvesting and cleaning of individual lines without cross-contamination, preparation, storage, and organization of several thousand bags of seed and their corresponding DNA samples can be laborious and require large amounts of space and resources. Proper storage is of immense importance as many seeds rapidly lose viability if stored under improper conditions. In addition, tracking a TILLING population and associated data over several generations and maintaining numbers on seed availability is greatly facilitated by establishing a database and bar-coding system. To assist groups that are new to TILLING, or are planning a new library, a flowchart called COAST (consider optimize achieve select TILLING) has been proposed by Wang et al. [21], providing a good starting point and helpful advice on launching a TILLING project.

The power of TILLING was first demonstrated in model systems such as *Arabidopsis* and *Drosophila *[24, 51], where it was shown that single mutations in specific genes could be identified. TILLING has later been successfully applied to a number of plant systems including barley, wheat, maize, rice, oat, pea, and soybean (Table 1). Thus, this technology provides the breeders with a new and sophisticated tool for crop improvement.

## 3. Mutant Discovery in TILLING Populations

### 3.1. Direct Sequencing

Direct sequencing using a Sanger-based method is the simplest method to screen a TILLING population, but it is also by far the most expensive one. DNA sequencing could be considered the “gold standard” for screening as all mutations can be easily identified. Although screening generally centers around one or a few genes, availability of a reference genome theoretically allows for assembly and analysis of complete mutant genomes. This can be particularly useful in cases where a phenotype is readily visible but no candidate gene has been identified. However, this also puts a great demand both on the speed and price of sequencing technologies (see Section 3.8).

### 3.2. Li-Cor

The most commonly used method to identify mutations in a TILLING population is by using the Li-Cor system (Table 1). It relies on the specific cleavage of mismatched bases formed as a result of repeated melting and reannealing of a PCR product amplified from a region of interest. If a mutation is present, a hybrid DNA molecule with a single mismatch will be generated. It is then selectively cleaved with an endonuclease, typically Cel-1 or Endo-1, producing two shorter fragments that can be separated by polyacrylamide gel electrophoresis [25]. By incorporating fluorescent dye-tags of different colours in the forward and reverse PCR primers, the amplified fragments can then be identified by the Li-Cor instrument. A single Li-Cor can run a 96 lane gel and the sensitivity is high enough to allow up to 16-fold pooling of samples, thus totaling 768 samples per run in diploid organisms. However, when screening large hexaploid genomes this number is reduced considerably due to the increased genomic complexity. In addition, there are a number of inherent drawbacks with the Li-Cor method that need to be considered. Parameters like fluorescent dye-primer- and DNA concentrations as well as the ratio between the cleavage enzyme and PCR product concentrations all affect the results and need to be optimised. In addition, for an efficient detection of the fluorescent fragments and acceptable throughput, a specialised instrument is required. On the other hand, the maximum length of amplicons using a Li-Cor system is as high as 1.5 Kb, among the longest of all methods. Both Endo-1 and Cel-1 are relatively expensive, but a protocol is available describing how to isolate Cel-1 directly from celery stalks [52]. The resulting enzyme extract, CJE (celery juice extract) can replace purified enzyme in many applications, substantially reducing the price per reaction. Several bioinformatic tools exist to help design primers for Li-Cor use, the most popular being CODDLE (http://www.proweb.org/coddle/) which combines primer functional analysis with an algorithm that, based on chosen mutagen and gene structure, identifies gene regions where deleterious mutations are most likely to occur. For postrun gel analysis, GelBuddy is an application that helps automate band detection in electrophoretic gels while ParseSNP can predict the expected effect of the introduced SNP on protein function.

### 3.3. High-Performance Liquid Chromatography (HPLC)

An HPLC-based method was used in early experiments with TILLING and can be considered as a sensitive option for screening [23]. Samples are treated with Cel-1 mismatch-cleave enzyme, as in the Li-Cor method and then separated using HPLC. A heterozygous mutation would appear as two new elution-peaks with the sum of their sizes equaling the original PCR product [24]. An 8-fold pool of samples is recommended in a diploid organism allowing 8 samples to be analyzed simultaneously, although diploid pools of up to 32-fold are possible [23]. However, running several samples concurrently would require the use of several HPLCs, limiting its potential as a high-throughput screening platform.

### 3.4. Electrophoresis

Regular electrophoresis using agarose or polyacrylamide (PAGE) gels has been proposed as a cheap alternative to Li-Cor systems for high-throughput screening. The protocols are based around the same mismatch-cleave system using Cel/endoenzymes but rather than fluorescent dyes, ethidium bromide (EtBr) is used to visualise the fragments after separation on an agarose gel. According to the authors, an 8-fold pool is possible with an upper amplicon length limit of 3 Kb [53]. This method has been used to successfully screen a wheat population for *waxy* and hard grain mutants using a 4-fold pool on thin (<4 mm) gels [36]. As agarose gel electrophoresis does not require any special equipment, and as Cel-1 can be replaced with celery juice extract (CJE), this may be the method of choice for low-budget TILLING [52]. However, due to the decreased sensitivity of the method compared to Li-Cor a larger amount of Cel-1 is required per sample, further stressing the need for home-made CJE.

### 3.5. Capillary Electrophoresis

Capillary electrophoresis (CE) can also be used to screen TILLING populations [32]. After cleavage with Cel-1/endo-1 the sample is mixed with EtBr, loaded into glass capillaries, and separated using electrophoresis. The presence of DNA is measured by UV-light excitation of DNA-bound EtBr at the end of the capillary and an absorption spectra over time is digitally generated. A mutated strand will add new peaks to the graph. The maximum fragment length is approximately 1.5 Kb, rivaling that of Li-Cor. The detection limit is also high enough to resolve an 8-fold pool [32]. An alternative method to CE is conformation sensitive capillary electrophoresis (CSCE) where, contrary to standard CE techniques, enzymatic degradation is not necessary [37]. In this method, PCR and melt-annealing are performed, as in other methods, but Cel-1/Endo-1 is not added. The capillary is instead loaded with a semidenaturing gel (CAP), capable of separating homoduplexes from heteroduplexes as the “kink” caused by a mismatch affects migration rate. Using this method, an 8-fold pool of diploid DNA is possible, although the authors themselves recommend a 4-fold pooling [37]. All types of capillary electrophoresis suffer from a slight decrease in sensitivity owing to the use of intercalating dyes rather than fluorescent primers. However, analysis is very fast, around 5–10 minutes per run and the instrument can be upgraded to handle 96 lanes concurrently. The downside of CE is the high instrument cost requiring a substantial initial investment.

### 3.6. High-Resolution Melt (HRM)

In HRM, intercalating dyes are used that fluoresce only when bound to DNA. When the temperature is gradually increased, DNA-strands will melt apart causing a release of the dye and the total fluorescence will decrease in a predictable way. The results are displayed as temperature/fluorescence graphs. A mutation will cause a shift in the graph as the mismatched base changes the melting temperature. Heterozygotes are easily identified by comparison of normalised melting curves with those of homozygotes or wild-type samples [54, 55]. Though sensitive, HRM is limited by both amplicon GC content and length, a typical read only covering 150–500 bp, which is much shorter than Li-Cor and CE. HRM is especially useful when a specific region with known impact on protein structure is the target or when the gene of interest contains many short exons and thus a short read length is acceptable. A drawback is that specialised software has to be used to interpret the different melt-curves. HRM can be performed on standard qPCR-machines with a simple software upgrade and is thus a suitable platform for initial TILLING screenings. HRM has been successfully applied in identification of mutations in wheat [56], *Medaka *[57], tomato [37], and *Arabidopsis *[43].

### 3.7. MALDI-TOF

Matrix-assisted laser desorption ionization time-of-flight (MALDI-TOF) spectroscopy has, since its inception in 1985, become a mainstay tool for analysis in the fields of polymer chemistry and proteomics. MALDI-TOF has also found some use in the field of high-throughput SNP discovery. However MALDI-TOF has not yet been fully exploited in SNP discovery and there is currently only one standardised, high-throughput method available, developed by SEQUENOM and known as MassCleave [58]. This method uses a synthesis step by T7-R&DNA polymerase followed by RNAse degradation to generate small RNA fragments that can be detected by the instrument. Once detected, the fragments can be reassembled *in silico* to provide a picture of the screened PCR product and to pinpoint mutations.

Recently, a new matrix of diaminobenzophenone (DABP) was introduced, for the analysis of nucleotides. Compared to traditional 3-HPA (3-hydroxy piccolinic acid), DABP has a 100-fold greater salt tolerance while retaining a similar resolution and sensitivity [59]. This matrix could therefore be a simple and elegant alternative to 3-HPA in SNP analysis, as the presence of even small concentrations of K+ and Na+-ions in the sample solution severely affects the sensitivity of the assay. Compared to Li-Cor-based techniques, MALDI-TOF is relatively straightforward. The enzymatic degradation steps are simple and robust and do not require optimization of individual steps or titration of the enzymes used. The method is also very sensitive and is capable of identifying heterozygote mutations in a hexaploid organism. Another potential benefit is that the method does not rely on heteroduplex formation, allowing for accurate detection of homozygous mutations without the need to pool samples. In fact, a homozygous mutation would be more visible as it leads to the disappearance of a mass peak in the MALDI graph. In extension, this means that MALDI-TOF-based screenings are even more relevant in late-stage TILLING populations where an increasing amount of mutations are homozygous. A proof-of-concept screening was published using the original protocol for MALDI-TOF based SNP discovery [22]. 

**Figure 1:**
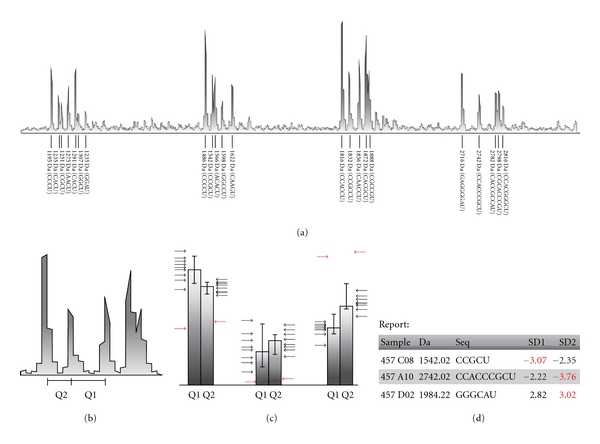
An overview of mutant identification using MALDI-TOF. (a) Each identified peak is matched to an expected peak. (b) Each peak is compared to the preceding and succeeding peak in the graph and two quotas are calculated and stored. (c) A sample-set-wide mean and standard deviation is calculated for each peak set and compared to the standard deviation of each individual sample peak (arrows). Outliers above a preset threshold are flagged as “suspicious” (red arrows). (d) Data is presented in a table as well as a colour-coded sequence (not shown).

We adjusted and optimised the SEQUENOM MALDI-TOF protocol for TILLING applications by decreasing reaction size, changing to a more salt-tolerant DABP matrix, and developing software for automated screening of samples. In our modified protocol, reaction size was halved and only 1/8th of the original enzyme amount was used without loss in sensitivity. Additionally, we developed a new software to accurately identify new SNPs (Figure 1). 

While waiting for more economical alternatives, TILLING screening using MALDI-TOF instruments could be a good complement to other screening methods and even as an alternative to large investments in Li-Cor technology. This is especially true for those laboratories where MALDI-TOF equipment, with its myriad of uses, is already part of the basic infrastructure.

### 3.8. Emerging Technologies

Next-generation sequencing (NGS) has significantly accelerated the prospects of identifying mutations at the whole-genome level. Decreasing sequencing costs due to improved technical accuracy, improved throughput, and increased capacity compared to only a few years ago has led to a great potential for NGS in TILLING. The two most commonly used NGS platforms are the 454 GenomeSequencer FLX Ti (Roche Applied Science) and the Illumina (Solexa) Genome Analyzer. While the average read length for 454 is 750 bases, Illumina only gives up to 100 bases per read but in turn generates a much greater amount of sequence data. In addition, these technologies are under constant development both with regard to read length, data quality and the number of sequences generated. As an example, Roche has recently implemented up to 1 Kb read lengths with the GS-FLX+ system.

There are already several proof-of-concept methods for applying NGS in TILLING applications. Using 3-dimensional pooling it is possible to screen one or several genes of interest in a single FLX-454 run. Experiments suggest that as many as 12,000 samples may be analyzed simultaneously on a single 454-picotiter plate (PTP) using KeyPoint technology, as successfully tested on a tomato TILLING population [60]. Illumina sequencing has also been adapted to high-throughput TILLING, and has been used to screen bread-wheat, durum-wheat, and rice populations [61]. The method called CAMBa (Coverage Aware Mutation-Calling using Bayesian analysis), not only identified several mutations that had been missed by CJE mismatch-cleave based TILLING, but also confirmed already known ones with fewer false positives [61]. As the amount of data generated from NGS is immense, some knowledge of bioinformatics and access to computational resources are invaluable during analysis. In addition to already established techniques, a new technology based on single molecule sequencing, PacBio *RS* is now also available. Average read length for this instrument exceeds 1 Kb, more than 10% of reads are between 1.5 and 2.5 Kb while some reads are longer than 4500 bp [62]. With recent technical updates, the sequencer delivers approximately 35 Mb sequencing data per run. This technique will be especially useful for nonsequenced genomes where no prior alignment scaffold exists due to its impressive read lengths, but has yet to be adapted to TILLING. Aside from direct screening, NGS has also been used for SNP discovery. Recently, NGS was performed on 17 wild and 14 cultivated soybean genomes with an average coverage of 5x and greater than 90% depth. This work identified high allelic diversity of 205,614 tag SNPs that could be useful for QTL mapping and association studies [63]. A NGS study on six elite maize cultivars resulted in identification of 1,000,000 SNPs, 30,000 insertion-deletion polymorphisms, and presence/absence variation of several genes amongst the six lines [64]. These studies highlight the growing importance of high-throughput technologies in fields other than mutation screening.

## 4. From Genotype to Phenotype

Contrary to traditional screening methods done by plant breeders, TILLING focuses on first identifying mutations within genes of interest and then linking those mutations to a specific phenotype. However, this approach is only possible when a gene linked to the trait of interest is known and the gene sequence available. Using software and maps of conserved sequences within the gene it is then possible to predict which of the identified mutations that are most likely to cause changes in protein structure or aborted translation resulting in a nonfunctional product. The potential phenotypes identified in this way can then be verified by anatomical, histological, physiological, or biochemical studies. Although theoretically straightforward, there are several problems that might arise during the screening process and subsequent analysis. Since the screening takes place at the DNA-level, enhancer and promotor mutations that are upstream of the gene of interest can be difficult, if not impossible to detect unless a full genomic sequence is available, which is not the case for most nonmodel systems. Another complication stems from the fact that a single mutation, even if predicted to be deleterious does not necessarily affect overall cellular function. Homologs or paralogs of the gene of interest may still be expressed, leading to a low or nonexistent penetration of the mutation. This is especially true for hexaploid plants where a homolog of the gene of interest may exist in all three genomes and when one allele is mutated, two others may compensate for the loss. In practice it is therefore often necessary to identify knockout mutations in all alleles by laborious screenings followed by time-consuming crosses to stack the different mutations in the same genome. This can severely delay the development of the final trait.

Despite these drawbacks, several groups have reported successes in linking genotypic change to novel phenotypes in a variety of crops. Most noticeably in wheat, where traits related to the *waxy* phenotype [29, 36, 47] and grain hardness are being developed [36], in soybean where TILLING has proven useful in increasing the oleic acid content through the identification of mutations in the FAD1, 2, and 3 genes [65] and in *Sorghum* where lignin content has been decreased though mutation of COMT [35].

## 5. Identification of Novel Traits in Mutated Populations

### 5.1. Biochemical Screening

**Figure 2:**
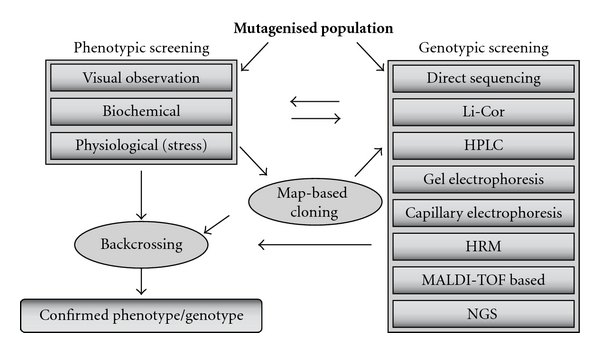
Overview of different methods to screen a mutagenised population and to develop a new stable character.

The main purpose of TILLING is to allow identification of mutations at the genetic level. However, this does not exclude the fact that TILLING populations, as well as other mutagenised populations also can be used for phenotypic screens. The principal difference between genotypical (TILLING) and phenotypical screening is illustrated in Figure 2.

**Figure 3:**
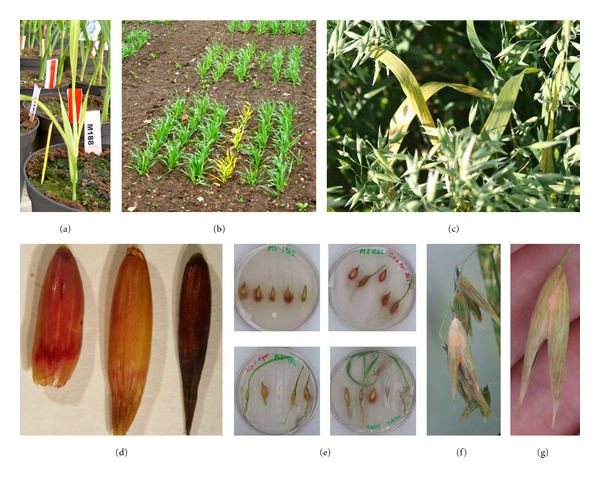
Examples of different phenotypes from an oat TILLING population. (a) Chlorosis marker from mutated line grown in the greenhouse. (b) Same marker grown in the field. (c) Same marker, clearly visible and stable in mature plants. (d) Phloroglucinol staining of oat cultivar Belinda (WT, left), a low lignin mutant (middle), and a high lignin mutant (right). Red coloration denotes presence of lignin. (e) Phenotypic screening for *Fusarium *resistance from random lines in the oat TILLING population. Seeds * *were placed on water agar and inoculated with ca 3000 spores of *Fusarium culmorum. *Upper left Petri dish shows the Belinda control. The remaining dishes show examples of resistant lines. (f) Examples of infected and noninfected microaxes in field grown plants. (g) Closeup of an infected microaxes.

Macromolecular composition and quantity of bioactive compounds like lignin and other fibers, lipid, and starch content are all quality characters that cannot be scored in the field. Lignin is found in secondary plant cell walls and provides rigidity to the plant. Lignin is considered a negative component in foragers as it blocks the digestion of cell-wall polysaccharides by microbial enzymes and is itself indigestible. Thus, crop varieties with lower lignin levels in the cell walls are preferred for feed since they are more energy efficient. A quick and economical assay for visually screening for altered lignin levels in seeds is the phloroglucinol-HCl assay (Wiesner test) [66]. We screened seeds from 1824 lines from an oat TILLING population [23] and identified 17 lines where the seeds had a reduced lignin stain intensity. For further confirmation, an acetyl-bromide method was then used for accurate quantification of lignin levels in the mutant seeds [22, 67, 68]. An example of the screen is illustrated in Figure 3(d).

Increased levels of dietary components that directly interfere with cholesterol absorption or excretion and thereby contribute to lowered plasma cholesterol levels are also very important breeding goals. One example is the mixed-linkage (1→3), (1→4) *β*-D-glucan soluble fibre which is mainly found in cereals and where oat and barley contain the highest concentrations. Using an assay kit available from Megazyme [69] we measured *β*-glucan content in seeds from 1500 random lines in an oat TILLING population [70]. We identified lines with increased levels of *β*-glucan as well as lines with levels less than half of what is found in Belinda, the original cultivar.

With the rising number of TILLING-populations (Table 1) we anticipate that these populations will be increasingly screened not only by TILLING, that is, genetic screening, but also by various advanced biochemical assays to identify important quality characters. Recently we set up an GC-MS assay and screened 1000 lines for *β*-sitosterol content and, in this relatively small sample size, identified lines with almost twice the normal levels. The advantage of a screen at the phenotypic level is that the target character is directly identified. The disadvantage, compared to a genotypic screening is that the specific mutation(s) mediating the phenotype remains undiscovered. There are several other examples from the literature elegantly demonstrating the power of biochemical screens [71–73].

### 5.2. Physiological Screening

Fungal pathogens represent a major threat to global agriculture. Global climate change with mild winters and higher humidity is expected to increase the problem even further. One particularly troublesome pathogen with high relevance in North America and Europe including Sweden is *Fusarium *[74]. Comprised of more than 1000 different species, *Fusarium* cause diseases in major agricultural crops like wheat, barley, maize, and oats. In addition, *Fusarium sp.* also produce a plethora of mycotoxins which accumulate in the grain, enter the food chain, and pose serious threats to human and animal health. A particular challenge is *Fusarium* head blight disease (FHB), for which there are currently no satisfactory management strategies available and where fungicide treatments give mixed and unpredictable results, sometimes even worsening mycotoxin contamination [75]. Unfortunately, the variation in the breeding populations does not seem to be high enough to identify and develop lines resistant to the disease.

On the other hand, even for characters that vary considerably with environmental factors, like pathogen resistance, mutagenised populations could be used to identify resistant lines with a strong genetic component. The trick is to design an *in vitro* assay with such a stringent selection that single rare lines with strong resistance against the disease can be identified. We tested this concept by designing a petri dish assay to identify *Fusarium*-tolerant oat from a mutated population with a high variety [22]. We placed 5 seeds from each line of the oat TILLING population on water agar and inoculated each seed with approximately 5000 spores of *Fusarium culmorum*. Since the spores have difficulties developing on the water agar they instead germinate on the seeds and the growing fungi, in turn hindering seed germination. As can be seen in Figure 3(e), this infection is efficient and the selection is therefore extremely harsh. We screened 1300 lines and identified 63 lines that germinated despite the presence of the fungi. We graded the lines as moderately resistant, if at least one seed germinated and developed rudimentary roots and shoots, and resistant if several seeds germinated and developed further (Figure 3(e)). We then tested the best lines in the field by sowing 60 seeds in three rows of 20 seeds randomly distributed and interrupted by rows of three market varieties from Lantmännen SW Seed AB. At the two leaf stages, all plants in the field were sprayed with a mixture consisting of four different subspecies of *F. culmorum* and three of *F. graminearum*. The plants were watered regularly during the whole growth season to facilitate infection. The degree of infection was scored later in the season as pink pigment formation on the microaxes (Figures 3(f) and 3(g)). Out of 43 lines, 26 were less infected than the most resistant commercial variety and all but three showed a higher resistance than the original Belinda cultivar. Thus, this preliminary experiment seems very promising and indicates that phenotypic screening of mutagenised populations could be used to identify complex characteristics like pathogen resistance if the screening method is carefully designed.

## 6. From Phenotype to Genotype

To be truly useful, identification of a strong genetic character in a mutagenised population by a phenotypic screening procedure should be followed by a characterization of the molecular event underlying the modified character. In plants with sequenced genomes, that is, where reference sequences are available, novel phenotypes can be characterised using a combination of whole-genome resequencing, linkage maps, and microarrays, providing a comprehensive picture of gene expression changes and newly introduced SNPs compared to wild-type specimens. A classical example is the identification of a GA20 oxidase mutation as a cause for the semidwarf phenotype used in many commercial rice varieties. Using genetic maps, the trait was linked to a region of chromosome 1. Combined with the knowledge that the dwarf phenotype had reduced levels of gibberellic acid (GA), a putative GA gene in that area was identified and sequenced using the rice reference genome as a base. The sequence showed a 280 bp deletion resulting in an inactive protein, explaining the decreased GA-levels [76]. Microarray technology has also been successfully applied in rice and *Arabidopsis *to connect genome-wide variations to specific phenotypes [77, 78]. However, next-generation technologies such as Illumina sequencing now outperform the more traditional microarray methods for SNP identification [79]. In one such approach, EMS-induced *Arabidopsis* Col-0 mutants with slow growth and light green leaves were screened to identify the causative mutations. The recessive mutants were first crossed with the *Landsberg erecta* ecotype. DNA from 500 F_2_ individuals was then pooled and sequenced using Illumina sequencing to up to 22-fold genome coverage. A software called SHOREmap was then developed to identify the mutations in the segregating population. The software detected a mutation causing serine to asparagine nonsynonymous codon change in the AT4G35090 gene [80]. In yet another approach, Austin et al. identified three genes involved in cell wall biosynthesis. They first screened the *Arabidopsis* EMS-treated Col-0 mutants for sensitivity to flupoxam that were previously known to affect cell wall assembly or integrity. The mutants were then crossed to *Landsberg erecta* ecotype. The genomic DNA was extracted from the F_2_ population and screened using Illumina GA sequencing. Through an in-house developed statistical approach, they were able to correctly identify the causative mutations and hence the genes responsible for the phenotype [81].

Since a mutation does not necessarily need to be in an exon of the candidate gene, identifying a mutation may be difficult if a reference genome is unavailable. Mutations such as promotor mutations, mutations changing genome structure, mutations upstream in the regulation pathway, and various micro-RNA mutations can all be responsible for the downstream effect. When a reference genome is not available, these factors can be extremely difficult and time-consuming to evaluate comprehensively. In such cases, an initial approach would be to obtain as many mutants as possible and evaluate each one separately, re-sequencing all genes of interest and performing qPCR experiments to gauge any possible changes in expression among the candidate genes. Although difficult, it is not impossible to obtain a genotype-phenotype association this way. Using EST libraries instead of the fully sequenced genome, Feiz et al. linked wheat grain hardness to *Puroindoline a *and *b *mutations in an EMS-mutagenised population [82]. A major caveat is that a link between genetic maps and genes are unknown in many cases, thus effectively robbing the researchers of a valuable selection tool for limiting the number of candidate genes.

## 7. Introgression of Stable Markers to Breeding Populations

Even though present elite cultivars are genetically fairly homogeneous, phenotypic differences between individual plants can always be seen in the field due to varying environmental factors. Cultivars grown at different sites with different fertilisation, pest and weed control regimes, weather conditions, and so on will exhibit differences not only in general plant architecture but also in quantity of specific macromolecules and metabolites. However, the influence of the environmental factor varies with the mechanism by which each particular mutation mediate the phenotype. Thus, if the genetic factor is strong for a specific trait, the variation in the expression of the trait will be smaller.

Examples of genetically strong and visible characters are leaf shape, colour, and presence of pubescence on the leaves or stems since these do not change noticeably with the environment. Such characters are therefore used as markers to distinguish market varieties from each other. In the ideal case such a visible, stable trait can also be correlated to a more specific, but invisible quality character. The experienced breeder could then score the quality character directly in the field even at varying environmental conditions. The key to a good selection strategy therefore involves the identification of environmentally stable phenotypes that correlate to a specific genotype.

However, often such correlations cannot be found for important quality characters like high fat, starch or protein content, fibre composition, reduced levels of toxic compounds, and enhanced postharvest processing properties. To identify these traits, more specific assays have to be performed. The drawback is that such assays often are time consuming and expensive and cannot be performed on a large number of samples.

On the other hand, if a mutagenised population with a very high variation is used, the probability of identifying a specific trait is increased and the number of assays needed to identify a certain quality character is decreased. In addition, the probability of finding rare mutations knocking out transcription factors or other pleiotropic genes is increased. Such mutations will have a stronger penetration and the corresponding phenotype will be less affected by outer, environmental parameters. As an illustration of the principle, Figures 3(a)–3(c) shows a chlorotic line identified in the greenhouse in an oat TILLING population [22]. In this particular mutation, the genetic factor is strong enough to be easily detected by the naked eye during the entire growth season. Of course, nonvisible mutations that can only be detected biochemically can, in an analogous way, still be genetically strong.

Once identified in a mutagenised population and tested for genetic stability in the field, the character can be introgressed into breeding lines lacking that character. Ideally, introgression should be done by the help of a marker since it reduces the number of necessary crosses and also ensures that as many random mutations as possible are eliminated from the mutagenised lines. Such a marker could be visible, biochemical, or molecular. A molecular marker, that is, a mutation or other DNA rearrangement that cosegregates with a useful quality character is preferable and has several advantages compared to conventional phenotypic selection. This is referred to as “molecular marker-assisted selection” (MMAS). Since MMAS is DNA based it is neutral and completely independent of environmental factors. Material for the assay can be collected from any tissue in the plant and at any developmental stage and the trait can often be scored very early in the plant growth cycle, even from seeds. This saves time, labour, and field space. Molecular markers can also be used to select for complex characters as long as the linkage to the marker is strong enough. If a molecular marker correlates to disease resistance, resistance can be scored without having to challenge the plant with the pathogen.

MMAS can be based on a mechanistic knowledge of how a particular mutation directly up-, downregulates, or completely knocks out a specific gene. In such a case it will be closely linked to a specific phenotype. However, MMAS could also be indirect, and based on a statistically significant link to the phenotype. Such markers are referred to as QTLs and could be single nucleotide polymorphisms (SNPs), microsatellite markers, or various DNA rearrangements that can be detected by DNA sequencing, PCR, Southern blot, MALDI-TOF, or other hybridization techniques. Semagn et al. [83] give an excellent review on various types of markers. Perhaps most importantly, MMAS can be automated and subjected to high-throughput screening. By automating DNA isolation, pipetting, separation, and evaluation using robots, fluorescent detection techniques, automatic scripts, and so forth, the screening procedure can be speeded up enormously and performed on a large number of markers in parallel.

## 8. Conclusion

During the last decade mutagenesis in breeding has again come of age. Plant mutagenesis, which increases the variation in crop plants that have been inbred for centuries, coupled with high-resolution genotypic or phenotypic screening methods allows breeders to select for traits that were very difficult to breed for only a few decades ago. The introduction of new genetic variation in inbred elite cultivars offers a unique possibility to identify novel traits, while retaining the agricultural excellence of the lines. With the rapid accumulation of genetic data from a wide range of crop plants, the continuous decrease of the costs associated with whole-genome sequencing, and the development of high-resolution analytical techniques, we have reached a point were we stand to gain both time and money by adding this toolbox to more traditional breeding techniques. Since markers are generated in the process, this approach also allows stacking of the useful characters, paving the way for the development of complex multigenic traits like abiotic stress resistance. Although still restricted to the capacity of the endogenous genome, mutagenesis and high-resolution screening will provide a very good complement to recombinant DNA technologies and genetically modified organisms (GMOs) in further development of crop plants that are better adapted to climate change and the increasing global population.

## Figures and Tables

**Table 1:** Published mutant populations in various plant species.

Species^a^	Year^b^	Mutagen^c^	Mutation rate^d^	Screening method^e^	Source^f^
*Arabidopsis*	2000	EMS	1/153 Kb	dHPLC, Li-Cor	McCallum et al. [24], Colbert et al. [25]
Rice	2001	DEB, GR, FN	1/40 Kb (deletion)	Phenotypic (stress)	Leung et al. [16]
*Lotus japonicus*	2003	EMS	1/502 Kb	Li-Cor, CE	Perry et al. [26, 27]
*Arabidopsis*	2003	EMS	1/208 Kb	Li-Cor	Till et al. [28]
Barley	2004	EMS	1/1 Mb	dHPLC	Caldwell et al. [23]
Maize	2004	EMS	1/485 Kb	Li-Cor	Till et al. [19]
Durum wheat	2005	EMS	1/40 Kb	Li-Cor	Slade et al. [29]
Bread wheat	2005	EMS	1/24 Kb	Li-Cor	Slade et al. [29]
Rice	2005	EMS	1/1 Mb	Li-Cor	Wu et al. [17]
Rice	2007	EMS	1/294 Kb	Li-Cor	Till et al. [18]
Rice	2007	Az-MNU	1/265 Kb	Li-Cor	Till et al. [18]
Pea	2007	EMS	1/669 Kb	Li-Cor	Triques et al. [30]
Soybean	2008	EMS	1/140 Kb	Li-Cor	Cooper et al. [31]
Soybean	2008	NMU	1/140 Kb	Li-Cor	Cooper et al. [31]
Rice	2008	MNU	1/135 Kb	CE	Suzuki et al. [32]
Barley	2008	Az	1/374 Kb	Li-Cor	Talamè et al. [33]
Rapeseed	2008	EMS	1/41 Kb	Li-Cor	Wang et al. [34]
Sorghum	2008	EMS	1/526 Kb	Li-Cor	Xin et al. [35]
Bread wheat	2008	EMS	1/23 Kb	AGE	Dong et al. [36]
Tomato	2009	EMS	1/735 Kb	CE, HRM	Gady et al. [37]
Barley	2009	EMS	1/500 Kb	Li-Cor	Gottwald et al. [38]
Cabbage	2009	EMS	1/447 Kb	Li-Cor	Himelblau [39]
Medicago	2009	EMS	1/424 Kb	CE	Le Signor et al. [40]
Medicago	2009	EMS	1/485 Kb	Li-Cor	Le Signor et al. [40]
*Arabidopsis*	2009	EMS	1/51 Kb	Li-Cor	Martín et al. [41]
Bread wheat	2009	EMS	1/40 Kb	PAGE	Uauy et al. [42]
Bread wheat	2009	EMS	1/41 Kb	Li-Cor	Uauy et al. [42]
*Arabidopsis*	2010	EMS	1/415 Kb	HRM	Bush and Krysan [43]
Melon	2010	EMS	1/573 Kb	Li-Cor	Dahmani-Mardas et al. [44]
Pea	2008	EMS	1/200 Kb	Li-Cor	Dalmais et al. [45]
Oat	2010	EMS	1/30 Kb	MALDI-TOF	Chawade et al. [22]
Tomato	2010	EMS	1/322 Kb	Li-Cor	Minoia et al. [46]
Bread wheat	2010	EMS	NA	Li-Cor	Sestili et al. [47]
*Brassica rapa*	2010	EMS	1/44 Kb	CE	Stephenson et al. [48]
Peanut	2011	EMS	1/931 Kb	Li-Cor	Knoll et al. [49]
Peanut	2011	DES	None detected	Li-Cor	Knoll et al. [49]
Sunflower	2011	EMS	1/475 Kb	Li-Cor	Sabetta et al. [50]

^a^Plant species used for developing the mutant population.

^b^Year of publication.

^c^Mutagen used for inducing mutations—EMS (ethyl methanesulfonate), Az-MNU (sodium azide plus methylnitrosourea), Az (sodium azide), DES (diethyl sulfate), GR (Gamma Ray Bombardment), FN (Fast Neutron Bombardment), and DEB (Diepoxybutane).

^d^Mutation frequency.

^e^Mutation screening method—CE (capillary electrophoresis), HRM (high-resolution melting), AGE (agarose gel electrophoresis), and PAGE (polyacrylamide gel electrophoresis).

^f^References.
